# Successful Treatment for Hypercalcemia due to Cosecretion of Parathyroid Hormone-Related Protein and 1,25-Dihydroxyvitamin D_3_ in Non-Small-Cell Lung Cancer: A Case Report and Literature Review

**DOI:** 10.1155/2020/2475725

**Published:** 2020-01-03

**Authors:** Takunori Ogawa, Jun Miyata, Koichi Fukunaga, Akihiko Kawana, Takashi Inoue

**Affiliations:** ^1^Division of Pulmonary Medicine, Department of Medicine, Keio University School of Medicine, Tokyo, Japan; ^2^Division of Infectious Diseases and Respiratory Medicine, Department of Internal Medicine, National Defense Medical College, Saitama, Japan; ^3^Department of Pulmonary Medicine, Sano Kosei General Hospital, Sano, Tochigi, Japan

## Abstract

Hypercalcemia of malignancy frequently manifests as paraneoplastic syndrome in patients with solid tumors. A 71-year-old man was diagnosed with stage IIIB lung squamous cell carcinoma. Laboratory examination revealed high serum calcium concentration with elevated serum parathyroid hormone-related protein (PTHrP) and 1,25-dihydroxyvitamin D_3_ levels. As the patient did not respond to the initial treatment with calcitonin, extracellular fluid infusion, and chemotherapy, systemic prednisolone was administered additionally. Thus, the levels of serum calcium normalized and PTHrP and 1,25-dihydroxyvitamin D_3_ decreased simultaneously. To our knowledge, this is the first case report on the successful treatment of hypercalcemia of malignancy caused by PTHrP and 1,25-dihydroxyvitamin D_3_ cosecretion in a patient with lung cancer.

## 1. Introduction

Hypercalcemia is a relatively common finding in cases of paraneoplastic syndrome. Hypercalcemia of malignancy (HCM) occurs in up to 20 to 30% of patients with cancer [1]. It can be classified into the following four types: caused by local osteolytic hypercalcemia, secretion of parathyroid hormone- (PTH-) related protein (PTHrP), secretion of 1,25-dihydroxyvitamin D_3_ (calcitriol), and ectopic hyperparathyroidism. PTHrP derived from solid tumors, especially squamous cell carcinoma, is a well-known mediator of HCM. However, calcitriol is rarely secreted from these tumors. Only few previous reports have described elevated levels of both blood serum PTHrP and calcitriol in patients with solid tumors [2–6]. Here, we report the case of a patient with squamous cell lung cancer, who developed hypercalcemia caused by cosecretion of PTHrP and calcitriol, which was improved by corticosteroids.

## 2. Case Presentation

**Figure 1:**
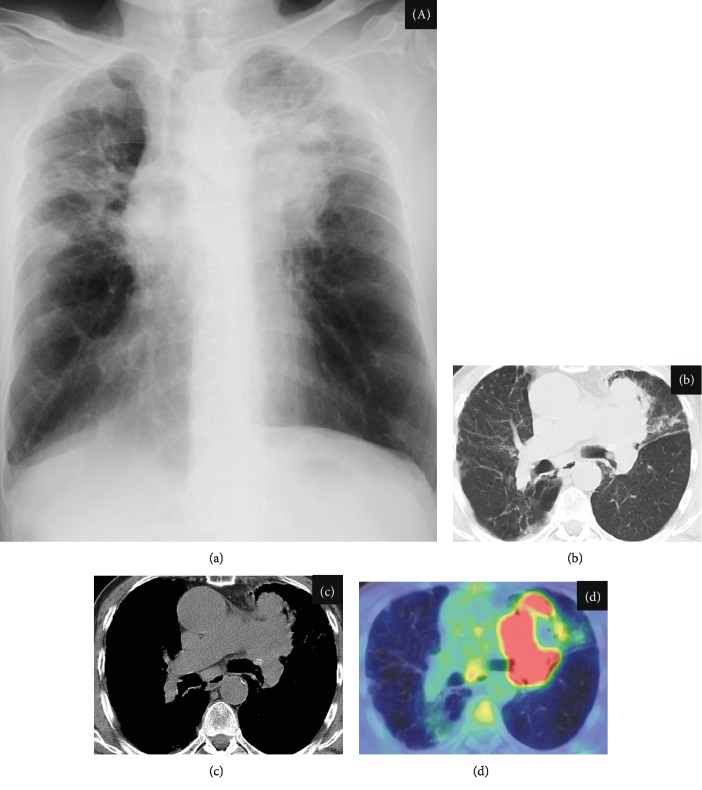
Chest radiography and F-2-deoxy-2-fluoro-D-glucose positron emission tomography/computed tomography (PET/CT) findings. (a) Chest radiography demonstrating a mass-like lesion on the left upper lung field and (b–d) PET/CT showing uptake of FDG by a left pulmonary hilar lesion mass and subcarinal lymph nodes.

**Figure 2:**
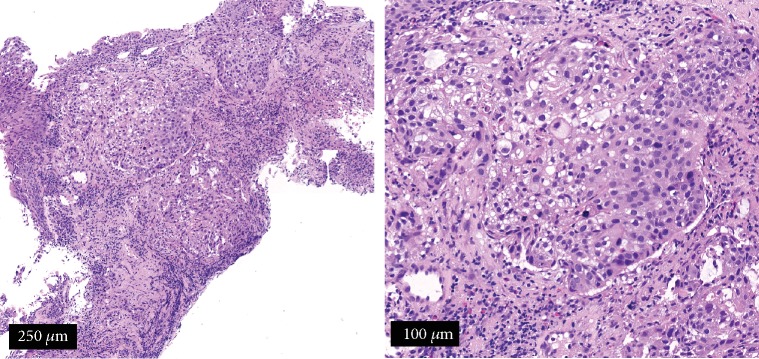
Pathological analysis of a transbronchial lung biopsy specimen using hematoxylin and eosin staining. Proliferation of large polygonal atypical cells with intercellular bridges is seen in the bronchial submucosa, suggesting squamous cell carcinoma. (a) Scale bar represents 250 *μ*m; (b) scale bar represents 100 *μ*m.

**Figure 3:**
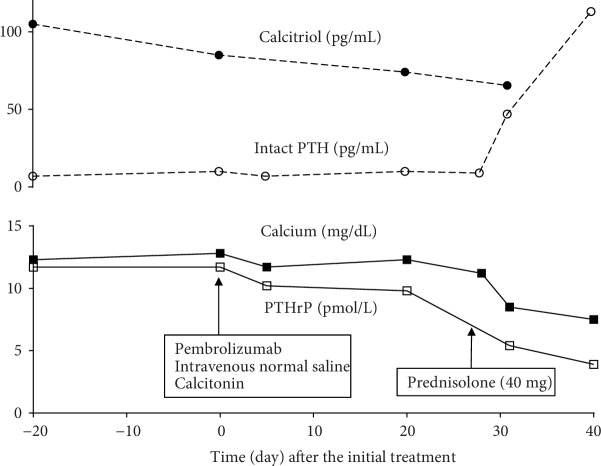
Clinical course of the present case. Calcitriol: 1,25-dihydroxyvitamin D_3_; PTH: parathyroid hormone; PTHrP: parathyroid hormone-related protein.

The patient was a 71-year-old man who presented with nausea, fatigue, and anorexia. He was referred to our hospital for investigation of these symptoms and abnormalities; as such, chest radiography was performed (Figure 1(a)). Laboratory examination revealed an elevated blood serum calcium level (12.3 mg/dL, serum albumin level 3.2 g/dL) and normal creatinine level, which did not indicate dehydration and renal dysfunction (Table 1). He did not take any hypercalcemia-inducing agents such as thiazide diuretics, theophylline, lithium, osteoporosis therapeutic drugs, and vitamin D supplements. Serum PTHrP level was elevated (11.7 pmol/L (reference value < 1.1 pmol/L)), although the intact PTH level was low (7 pg/mL (reference value 10-65 pg/mL)). Surprisingly, the serum calcitriol level was elevated (105 pg/mL (reference value 20-40 pg/mL)) although 25-OH vitamin D_3_ level was low (16 ng/mL (reference value > 20 ng/mL)). Integrated computed tomography and 18F-2-deoxy-2-fluoro-D-glucose (FDG) positron emission tomography (PET/CT) showed uptake of FDG by a left pulmonary hilar lesion and both ipsilateral mediastinal and subcarinal lymph nodes, because of which lung cancer with metastasis to the lymph nodes was highly suspected (Figures 1(b)–1(d)). No other organs, including the bone, liver, and bilateral adrenal glands, showed FDG uptake. Head magnetic resonance imaging revealed no metastatic lesions. Based on the results of a bronchoscopic examination showing proliferation of large polygonal atypical cells with intercellular bridges in the bronchial submucosa (Figure 2), the patient was diagnosed with stage IIIB lung squamous cell carcinoma (55% of tumor cells were positive for programmed cell death-ligand 1 expression). Lymphoma, granulomatous disease, and mycobacterial or fungal infections were ruled out based on the results of histopathological analysis. Pembrolizumab (200 mg/body every 3 weeks) was administered as the first-line treatment. In addition to anticancer treatment, calcitonin and intravenous 0.9% normal saline were first administered. Despite initial treatment for HCM, hypercalcemia did not improve. On day 28 from the initial treatment of HCM, systemic prednisolone (40 mg/day) was additively administered. Serum calcium level rapidly decreased thereafter and normalized (serum calcium 8.5 mg/dL, serum albumin 3.1 g/dL) on day 31. Serum PTHrP and calcitriol levels decreased simultaneously (PTHrP: 5.4 pmol/L; calcitriol: 65.4 pg/mL) in response to systemic prednisolone. Serum intact PTH levels were elevated to the normal range (47 pg/mL), indicating improvement of the negative feedback circuit to regulate serum calcium levels. However, lung cancer gradually progressed during two cycles of pembrolizumab, suggesting an inadequate therapeutic response to this immunotherapy. Respiratory failure developed, and the patient died on day 58.  Figure 3 shows the clinical course of this patient.

## 3. Discussion

To our knowledge, this is a rare case with cosecretion of PTHrP and calcitriol in lung cancer. There are only five previous reports of the cosecretion of PTHrP and calcitriol in cases of solid tumors including ovarian carcinoma, pancreatic neuroendocrine tumor, renal cell carcinoma, seminoma, and lung cancer [2–6], which are summarized in Table 2. In the previous report on lung cancer, the histological type was squamous cell carcinoma [6], as in our case. The patient could not receive systemic prednisolone because of rapid tumor progression with no improvement of hypercalcemia. However, systemic prednisolone could be effective for HCM induced by cosecretion of PTHrP and calcitriol in other cases with solid tumors. In summary, this is the first report of the successful treatment of HCM caused by cosecretion of PTHrP and calcitriol in a patient with lung cancer.

The mechanism of independently elevated PTHrP and calcitriol productions remains poorly understood. PTHrP secreted from solid tumors binds to the PTH-1 receptor causing hypercalcemia [7]. However, unlike PTH, it does not elicit calcitriol synthesis [8]. 1-*α*-Hydroxylase, an enzyme converting 25-OH vitamin D_3_ to calcitriol, normally expresses in the kidney. Previous report demonstrated that its expression in alveolar macrophages was higher in the lung cancer patients than in the healthy group [9]. Additionally, a human small cell lung cancer cell line constitutively expressed this enzyme [10]. These findings might explain the possible mechanism of PTHrP and calcitriol cosecretion in patients with lung cancer.

Hypercalcemia with an elevated calcitriol level has been reported in patients with some granulomatous diseases including sarcoidosis, tuberculosis, fungal infection, and lymphoma [11]. In our case, histopathological examination suggested no complications with these diseases. Thus, we concluded lung cancer as the cause of elevated serum calcitriol.

Treatment using extracellular fluid infusion, calcitonin, and chemotherapy was initially introduced in our case. Previous reports indicated that combined extracellular fluid infusion and calcitonin worked rapidly (within several hours) in some cases [12]. However, this therapy was not effective without responsiveness of the lung cancer to chemotherapy, which strongly suggested the necessity of an additive therapeutic drug.

In a case of metastatic renal cell carcinoma, prednisolone was chosen for the treatment of HCM, with beneficial effects [4]. Similarly, in our case, hypercalcemia showed a good response to systemic prednisolone as an add-on agent, possibly through steroid-mediated suppressive effects on the expression of enzymes necessary for PTHrP and calcitriol synthesis.

In four previous reports, bisphosphonates were selected for treating HCM due to cosecretion of PTHrP and calcitriol [2–4, 6], although hypercalcemia was not unaltered in the patients. Bisphosphonates were consequently unnecessary in our case because prednisolone therapy in addition to calcitonin and extracellular fluid infusion was sufficient to normalize the serum calcium levels. However, recurrence of HCM might be observed during initial therapy using prednisolone, though long-term observation was impossible due to the rapid tumor progression. Bisphosphonates could possibly be beneficial as additive agents in this situation [13, 14].

In summary, caution must be exercised by clinicians when patients with lung cancer show hypercalcemia with elevated serum levels of both PTHrP and calcitriol. Early introduction of prednisolone as an optimal therapeutic strategy should be recommended for patients with HCM induced by cosecretion of PTHrP and calcitriol.

## Figures and Tables

**Table 1:** Results of laboratory tests conducted on admission.

Value			Reference value
Peripheral blood			
White blood cells	11,900	/*μ*L	3,900-9,800
Neutrophils	85.8	%	
Lymphocytes	8.5	%	
Basophils	0.5	%	
Eosinophils	0.8	%	
Monocytes	4.4	%	
Hemoglobin	11.8	g/dL	13.5-17.6
Hematocrit	36.8	%	39.8-51.8
Platelets	413,000	/*μ*L	131,000-362,000
Blood biochemistry			
Total bilirubin	0.59	mg/dL	0.2-1.2
Aspartate transaminase	25	U/L	9.0-30
Alanine transaminase	17	U/L	4.0-35
Lactate dehydrogenase	197	U/L	80-260
Alkaline phosphatase	282	U/L	106-345
*γ*-Glutamyl transpeptidase	59	U/L	16-84
Total protein	7.5	g/dL	6.5-8.2
Albumin	3.2	g/dL	3.9-4.9
Urea nitrogen	16.4	mg/dL	8.0-20
Creatinine	0.94	mg/dL	0.6-1.1
Sodium	143	mEq/L	132-148
Potassium	4.2	mEq/L	3.6-5.0
Chloride	105	mEq/L	96-110
Calcium	12.3	mg/dL	8.2-10.2
Phosphorus	2.6	mg/dL	2.3-4.3
Intact PTH	7	pg/mL	10-65
PTHrP	11.7	pmol/L	<1.1
Calcitriol	105	pg/mL	20-40
25-OH vitamin D_3_	16	ng/mL	>20
Urine			
pH	5.5		5.0-9.0
Occult blood	(-)		
Sugar	(-)		
Protein	(-)		
Urea nitrogen	312	mg/dL	650-1,300
Creatinine	69.9	mg/dL	50-150
Sodium	38	mEq/L	70-250
Potassium	37.7	mEq/L	25-100
Chloride	41	mEq/L	70-250
Calcium	22.2	mg/dL	50-300
Phosphorus	36.8	mg/dL	<500

PTH: parathyroid hormone; PTHrP: parathyroid hormone-related protein; calcitriol: 1,25-dihydroxyvitamin D_3_.

**Table 2:** Summary of cases presenting cosecretion of PTHrP and calcitriol in solid tumors.

Author	Age	Sex	Primary tumor	Histological type	Tx of HCM	Sx	CTx	RTx	Course of the tumor	Outcome of HCM	Effective Tx of HCM
Hoekman et al. [2]	70	F	Ovarian carcinoma	Adenocarcinoma	Pamidronate	Yes	No	No	Resection	Improvement	Operation
					Hydrocortisone						
Van den Eynden et al. [3]	59	M	Pancreatic neuroendocrine tumor	Neuroendocrine tumor	Pamidronate	Yes	Yes	No	Reduction	Improvement	Chemotherapy
					Zoledronic acid						
Shivnani et al. [4]	57	M	Renal cell carcinoma	Clear cell	Pamidronate	No	Yes	No	Progression	Improvement	Prednisolone
					Prednisolone						
Rodriguez-Gutierrez et al. [5]	35	M	Seminoma	Seminoma	Calcitonin	No	Yes	No	Reduction	Improvement	Chemotherapy
Nemr et al. [6]	60	M	Lung cancer	Squamous	Calcitonin	No	No	No	Progression	No improvement	None
					Zoledronic acid						
					Furosemide						
Ogawa^a^	71	M	Lung cancer	Squamous	Prednisolone	No	Yes	No	Progression	Improvement	Prednisolone
					Calcitonin						

CTx: chemotherapy; HCM: hypercalcemia of malignancy; RTx: radiation therapy; Sx: surgery; Tx: treatment. ^a^Present case.
